# Subjective Memory Complaints Related to Satisfaction With Meaningful Activities Among Community-Dwelling Older Adults

**DOI:** 10.7759/cureus.73433

**Published:** 2024-11-11

**Authors:** Yoshihiko Akasaki, Takayuki Tabira, Michio Maruta, Hyuma Makizako, Suguru Shimokihara, Yuma Hidaka, Taishiro Kamasaki, Wataru Kukizako, Takuro Kubozono, Mitsuru Ohishi

**Affiliations:** 1 Department of Rehabilitation, Tarumizu Central Hospital, Kagoshima, JPN; 2 Department of Occupational Therapy, School of Health Sciences, Faculty of Medicine, Kagoshima University, Kagoshima, JPN; 3 Department of Occupational Therapy, Health Sciences, Nagasaki University Graduate School of Biomedical Sciences, Nagasaki, JPN; 4 Department of Physical Therapy, School of Health Sciences, Faculty of Medicine, Kagoshima University, Kagoshima, JPN; 5 Department of Occupational Therapy, School of Health Sciences, Sapporo Medical University, Sapporo, JPN; 6 Department of Rehabilitation, Sanshukai Okatsu Hospital, Kagoshima, JPN; 7 Department of Rehabilitation, Faculty of Rehabilitation Sciences, Nishikyushu University, Saga, JPN; 8 Department of Rehabilitation, Iida Orthopedic Clinic, Miyazaki, JPN; 9 Department of Cardiovascular Medicine and Hypertension, Graduate School of Medical and Dental Sciences, Kagoshima, JPN

**Keywords:** community based, meaningful activity, older adults, satisfaction, subjective memory complaints

## Abstract

Objective

This study explores the relationship between subjective memory complaints (SMC) and satisfaction with meaningful activities among community-dwelling older adults.

Methods

Data were analyzed from 539 older adults without cognitive decline (mean age 74.6 ± 6.1 years, 65.0% female) who participated in a community-based health check survey (Tarumizu Study, 2019). SMC was assessed using four questionnaires, and SMC was determined when at least one of the four questionnaires was applicable. From the Aid for Decision-making in Occupation Choice (ADOC), participants selected meaningful activities. Satisfaction with these selected activities was also evaluated. Satisfaction levels were compared between participants with and without SMC, and the relationship between SMC and satisfaction with meaningful activities was analyzed using multivariate analyses.

Results

The prevalence of SMC was 63.2% (n = 339). Participants with SMC reported lower satisfaction with meaningful activities compared to those without SMC (p = 0.012). Notably, participants who responded positively to SMC-2 (“Do you forget where you have left things more than you used to?”) (p = 0.027) and SMC-4 (“Do other people find you forgetful?”) (p = 0.004) had significantly lower satisfaction with meaningful activities. Logistic regression analyses revealed a significant relationship between SMC and satisfaction with meaningful activities after adjusting for covariates (OR, 0.79; 95% CI, 0.633-0.996; p = 0.046).

Conclusions

There is a relationship between SMC and satisfaction with meaningful activities among community-dwelling older adults. Participation in and satisfaction with meaningful activities may play a role in developing effective support for individuals with SMC.

## Introduction

The number of people with dementia is increasing rapidly as the world's population ages. Particularly, Japan has a high prevalence of dementia patients compared to other countries, making early detection and prevention of dementia increasingly urgent. Subjective memory complaints (SMC) have recently been recognized as a potential early indicator of dementia that precedes mild cognitive impairment (MCI) [1]. SMC is characterized by a subjective perception of memory impairment in the absence of objective cognitive decline as measured by neuropsychological assessment and psychometric tests [2]. Research has demonstrated that older adults with SMC, but without dementia, are at an increased likelihood of developing dementia and have a higher risk of mortality [3]. SMC is a prevalent issue among older adults, with 25%-50% of community-dwelling older adults experiencing SMC [2]. Addressing SMC could significantly impact the health maintenance of older adults, particularly in the early detection and prevention of dementia.

Engaging in activity is among the most promising strategies for older adults to promote healthy cognitive aging [4]. Activity has also been found to positively influence SMC. One study suggested that SMC is more severe in older adults who live with lower activity intensity [5]. In other studies, it has been reported that older adults with SMC are at an increased likelihood of withdrawing from activities like social and leisure activities [6]. Thus, previous studies have shown the relevance and effectiveness of activity in older adults in SMC. The World Health Organization (WHO) recommends that older adults engage in meaningful activities tailored to their individual characteristics to support healthy aging [7]. In other words, it is important that activities in the older adults consider psychological needs, such as personal characteristics and needs. Meaningful activities are those that meet basic psychological needs such as engagement, relevance to interests and past roles, and sense of belonging [8]. They provide individuals with a sense of purpose, aid in achieving personal or cultural goals, and contribute to overall life satisfaction [9]. These activities can range from daily routines to work, caregiving, social activities, and leisure activities [10]. Participating in meaningful activities is crucial for successful aging and can improve or maintain the mental and psychological function of older adults [7]. Recent research suggests that it is important to consider not only participation but also satisfaction with meaningful activities, as meaningful activities are evaluated from a subjective perspective. Satisfaction with meaningful activities is related with reduced depressive symptoms and higher life satisfaction [11,12]. Thus, older adults can benefit a lot from participating in and being satisfied with meaningful activities that take into account their individual characteristics and needs.

There is some consensus regarding the relationship between meaningful activity and cognitive function. Participation in meaningful activities is effective approach to dementia [13]. Furthermore, engaging in meaningful activities may delay cognitive decline in MCI people [14]. Thus, meaningful activity has been reported to be associated with objective cognitive function. However, the relationship of meaningful activities in the preclinical phase, such as SMC, which is prior to dementia and MCI, is not clear. The perception of cognitive decline can deter adults with MCI from participating in meaningful activities and is likely to reduce their satisfaction with these activities [15]. Subjective perceptions of memory impairment, such as SMC in older adults, are therefore likely to be associated with meaningful activities and satisfaction with them. It is important to investigate the association between meaningful activity and satisfaction with SMC in order for older adults to practice prevention of future cognitive decline through activity.

Therefore, this study aimed to explore the relationship between SMC, meaningful activities, and satisfaction with these activities among community-dwelling older adults. Our hypothesis posited that SMC is related with satisfaction derived from meaningful activities, irrespective of the specific activity categories among community-dwelling older adults. The findings from this study could potentially inform the development of effective interventions to support older adults experiencing SMC.

## Materials and methods

Participants

**Figure 1:**
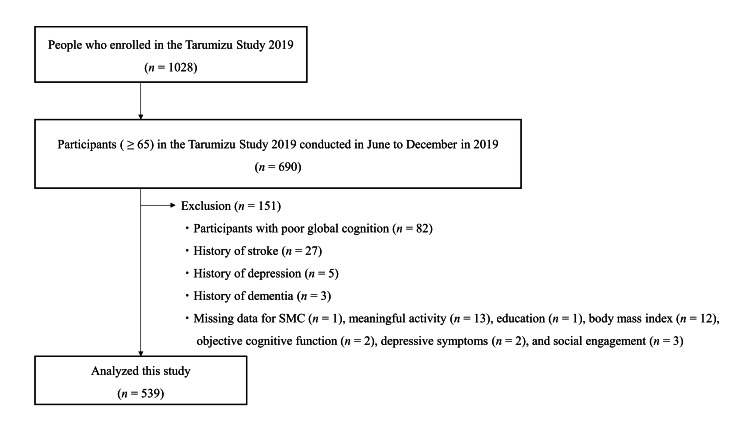
Flowchart outlining the inclusion and exclusion criteria employed in this study

This cross-sectional study used data from community-dwelling older adults aged 65 years and older who participated in the Tarumizu study. This study is a community-based health examination conducted in Tarumizu City, Kagoshima Prefecture, Japan, where the aging population exceeds 40%, in collaboration with Kagoshima University Faculty of Medicine, Tarumizu City Hall, and Tarumizu Chuo Hospital since 2017. For this study, we analyzed data from the health examination survey conducted between June 2019 and December 2019. This study employed stratified sampling to ensure the appropriate inclusion of older adults within the population. Specifically, we initially recruited residents aged 40 years and over living in Tarumizu City during the survey period, using invitations sent by postcard, city newsletters, and online announcements. Within this broader age group, we then selected individuals aged 65 years and older for analysis, as this age subgroup was the primary focus of the study. Among the 1,028 recruited participants, 690 individuals aged 65 years or older were included in our analysis. The exclusion criteria were (1) participants with low global cognitive function (Mini-Cog test score < 3, n = 82); (2) history of neurological or mental conditions, including stroke (n = 27), depression (n = 5), and dementia (n = 3); and (3) incomplete data (n = 34). Following these exclusions, the final sample comprised 539 community-dwelling older adults aged 65 years or older (mean age 73.6 ± 6.1 years, 65.0% female) (Figure 1). The ethics committee of the Faculty of Medicine, Kagoshima University approved the study protocol (Ref No. 170351, approval date: October 26, 2018), and informed consent was obtained from all participants before they were included in the study.

Operational definition of SMC

Although several methods exist for assessing SMC, Tsutsumimoto’s questionnaire was used in this study to assess SMC [16]. This questionnaire was based on the Cambridge Mental Disorders of the Elderly Examination questionnaire and the Subjective Memory Complaints Scale and has been used in a previous large-scale study in Japan [16]. Participants were considered to have SMC if they answered affirmatively to any of the four questions: SMC-1 (Do you have any difficulty with your memory?), SMC-2 (Do you forget where you have left things more than you used to?), SMC-3 (Do you forget the names of close friends or relatives?), and SMC-4 (Do other people find you forgetful?). The questionnaire is available in the appendix.

Meaningful activities' assessment

Meaningful activities were defined as “activities that individuals consider important in their daily life” in this study [11]. Meaningful activities were assessed using the Aid for Decision-making in Occupation Choice (ADOC) iPad application (Apple, Cupertino, CA, USA) [17]. The ADOC was created to identify activities that are meaningful to clients undergoing rehabilitation. The ADOC includes activities listed in the International Classification of Functioning, Disability and Health, such as self-care, mobility, domestic life, work/education, interpersonal interaction, social life, sport, and leisure. Data on meaningful activities were collected through face-to-face interviews using the ADOC. Initially, participants were briefed by the researcher on the definition of meaningful activities. Subsequently, participants chose up to 20 activities from the ADOC that they considered meaningful in their life. From these, they then selected up to five activities they deemed particularly meaningful based on their importance and priority in their current daily life. Finally, participants rated their satisfaction with each activity on a scale from 1 to 5 (1 being very dissatisfied and 5 being very satisfied). Previous studies have validated the reliability of the ADOC satisfaction measure [12]. Participants also rated their ability to perform each of the meaningful activities they selected on a scale from 1 to 10 (1 indicating great difficulty and 10 indicating perfect performance). In this study, we focused on analyzing the activities that participants considered most meaningful.

The evaluators in this study were occupational therapists and occupational therapy students who received training, including two two-hour lectures and a 30-minute practical session, before the survey was conducted. The study was conducted 12 times over one year, with researchers providing the same educational material each time.

Covariates

Data were gathered on sociodemographic variables including age (years), sex, education (years), body mass index, and living arrangements, alongside assessments of objective cognitive function, physical frailty, depressive symptoms, and social engagement, as potential factors influencing SMC [18].

In this study, objective cognitive function was assessed using the National Centre for Geriatrics and Gerontology-Functional Assessment Tool (NCGG-FAT). This tool evaluates four domains: memory (immediate recognition with Word List Memory I and delayed recall with Word List Memory II), attention (tablet version of the Trail Making Test (TMT) part A), executive function (tablet version of the TMT Part B) and processing speed (tablet version of the Symbol Digit Substitution Task) [19]. The NCGG-FAT demonstrates moderate to high validity, high test-retest reliability in community-dwelling older adults.

Physical frailty was determined by the five criteria established by Fried: slowness, weakness, exhaustion, low physical activity, and weight loss [20]. Each criterion was based on previous studies and was as follows [21]: Slowness was assessed by measuring maximum grip strength, with thresholds set at less than 26 kg for men and less than 18 kg for women. Exhaustion was indicated by a positive response to the question, "Have you ever felt tired for no reason in the past two weeks?." Low physical activity was identified by negative responses to both questions: (1) "Do you do any light exercise or sports for your health?" and (2) "Do you do any regular exercise or sports?." Weight loss was defined by a positive response to the question, "Have you lost more than 2 kg in the last six months?." Participants exhibiting three or more of these components were classified as physical frailty.

Depressive symptoms were assessed using the 15-item Geriatric Depression Scale (GDS15), with a total score of 5 or higher indicating depressive symptoms [11,22].

Social engagement was evaluated by the components of the Japan Science and Technology Agency Index of Competence [23]. This evaluation was based on an individual’s participation in neighborhood associations, regional events, charitable activities, and their role in a residents’ association (0, no; 1, yes). The scores were summed to produce a total score ranging from 0 to 4, with higher scores reflecting greater social engagement.

Statistical analyses

Data were analyzed using Pearson's χ^2 ^test for categorical variables and Student's t-test for continuous variables to compare characteristics between participants with and without SMC. Effect sizes were calculated using Cramer's V and Cohen's d to determine the magnitude of the differences.

The associations between SMC and satisfaction with meaningful activities were examined through logistic regression analyses using three models: a crude model, adjusted model 1, and adjusted model 2. In all models, the presence of SMC was the dependent variable. The crude model included only the presence of SMC as an independent variable. Adjusted model 1 incorporated sociodemographic variables as covariates, while adjusted model 2 included sociodemographic variables, objective cognitive function, physical frailty, depressive symptoms, and social engagement as covariates. Multicollinearity was checked using the variance inflation factor (VIF), which showed no concerns (VIF < 2.0).

To determine which SMC questions were associated with satisfaction with meaningful activities, satisfaction levels for each SMC question (SMC-1, SMC-2, SMC-3, and SMC-4) were analyzed using Student's t-tests. All statistical analyses were conducted using EZR version 1.55, with a p-value of < 0.05 considered statistically significant.

## Results

Participants characteristics

**Figure 2:**
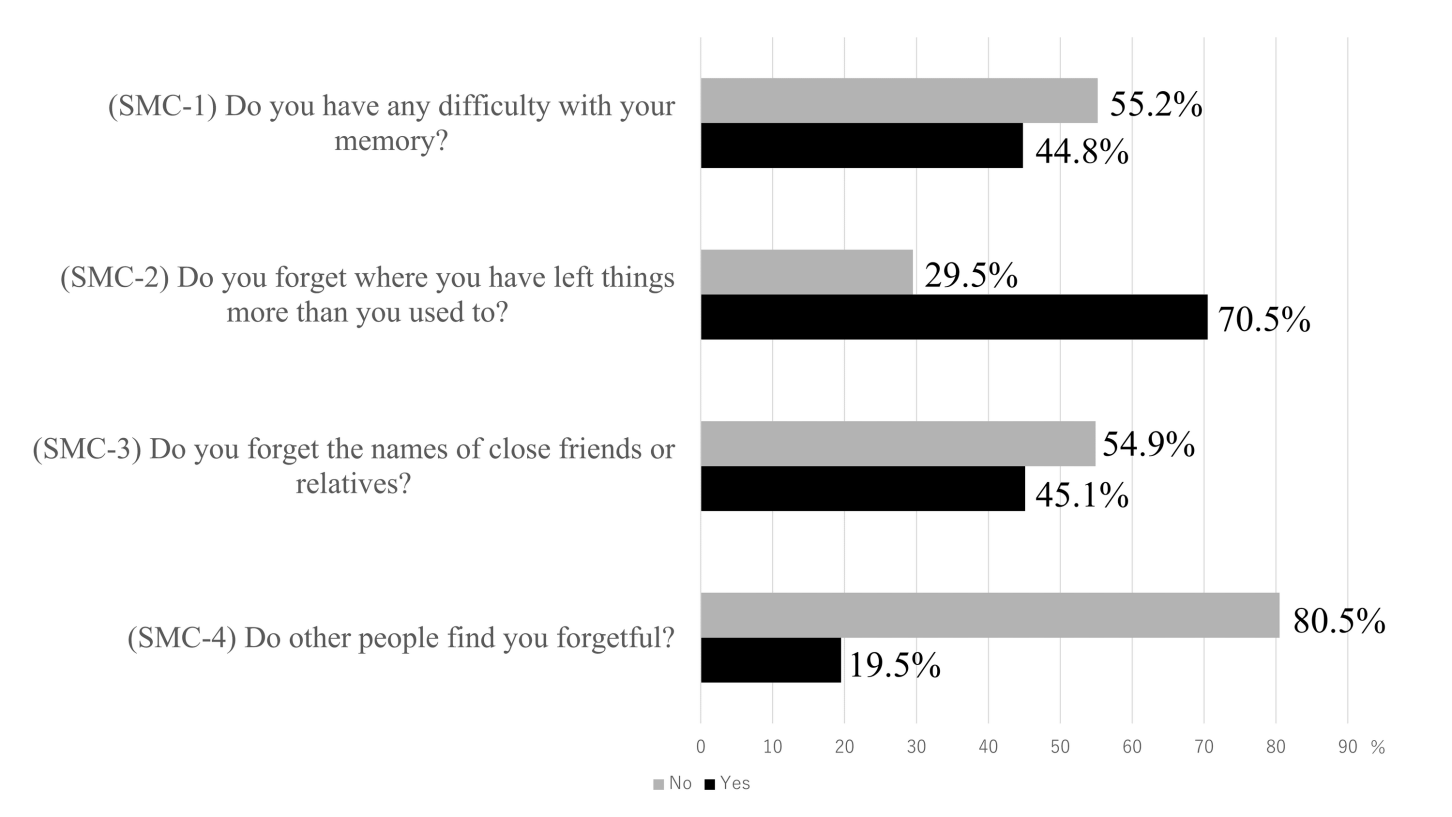
Status of responses to each question SMC: Subjective memory complaints This figure shows the four questions on SMC and the status of responses to each

Table 1 presents the characteristics of the study participants. A total of 539 older adults were included in the analyses. Among these participants, 339 (63.2%) were classified as having SMC. SMC-2, "Do you forget where you have left things more than you used to?" recorded the highest number of responses in the SMC group (Figure 2). Participants with SMC reported significantly lower satisfaction with meaningful activities compared to those without SMC (p = 0.012). However, there were no significant differences observed in the performance (p = 0.084). Additionally, participants with SMC exhibited poorer memory skills (p = 0.002), a higher prevalence of physical frailty (p = 0.024), and more depressive symptoms (p < 0.001) compared to those without SMC.

**Table 1:** Participants characteristics SMC: Subjective memory complaints; SD: standard deviation Statistical tests performed:^ †^Student’s t-test; ^‡^Person’s χ^2 ^test

	All participants (n = 539)	Without SMC (n = 200)	With SMC (n = 339)	Effect size	p-value
Age (years), mean (SD)	73.6 ± 6.1	73.0 ± 5.9	74.0 ± 6.3	0.171	0.056 ^†^
Female, n (%)	344 (63.8 %)	128 (64.0 %)	216 (63.7 %)	0.003	0.947 ^‡^
Educational history, (years), mean (SD)	11.6 ± 2.2	11.7 ± 2.1	11.5 ± 2.3	0.101	0.259 ^†^
Body mass index, mean (SD)	23.1 ± 3.2	23.1 ± 3.4	23.1 ± 3.1	0.170	0.956 ^†^
Living alone, n (%)	150 (27.8 %)	52 (26.0 %)	98 (28.9 %)	0.031	0.467 ^‡^
Objective cognitive function, mean (SD)					
Attention (seconds)	21.3 ± 7.6	20.7 ± 8.1	21.7 ± 7.2	0.133	0.137 ^†^
Executive function, (seconds)	43.9 ± 32.1	41.4 ± 34.1	45.5 ± 30.8	0.127	0.154 ^†^
Processing speed, (score)	44.3 ± 10.8	45.2 ± 9.9	43.7 ± 11.2	0.134	0.121 ^†^
Delayed recall, (score)	4.8 ± 2.1	5.1 ± 2.0	4.6 ± 2.2	0.265	0.002 ^†^
Physical frailty, n (%)	33 (6.1 %)	6 (3.0 %)	27 (8.0 %)	0.100	0.024 ^†^
Depressive symptom, n (%)	106 (19.7 %)	18 (9.0 %)	88 (26.0 %)	0.206	< 0.001 ^‡^
Social engagement (points), mean (SD)	73.0 ± 5.9	2.7 ± 1.4	2.6 ± 1.4	0.014	0.872 ^†^
Satisfaction with meaningful activity, mean (SD)	4.3 ± 0.8	4.5 ± 0.8	4.3 ± 0.9	0.110	0.012 ^†^
Performance with meaningful activity, mean (SD)	8.4 ± 2.1	8.6 ± 2.0	8.3 ± 2.2	0.070	0.083 ^†^

Relationship between SMC and satisfaction with meaningful activities

Table 2 displays the logistic regression models examining the relationships between SMC and satisfaction with meaningful activities. After adjusting for potential covariates, satisfaction with meaningful activities remained significantly associated with SMC (adjusted model 1: OR, 0.74; 95% CI, 0.599-0.925; p = 0.008; adjusted model 2: OR, 0.79; 95% CI, 0.633-0.996; p = 0.046).

**Table 2:** Relationship between SMC and satisfaction with meaningful activities OR: Odds ratio; CI: confidence interval; VIF: variance inflation factor In each model, the presence of SMC was set as the dependent variable (0 = without SMC; 1 = with SMC). Crude model: the presence of satisfaction with meaningful activities was set as the independent variables. Adjusted model 1: the presence of satisfaction with meaningful activities was set as independent variables and adjusted using demographic variables (age, sex, education, body mass index, and living status) as covariates. Adjusted model 2: the presence of satisfaction with meaningful activities was set as the independent variables and adjusted using demographic variables, objective cognitive function, depressive symptoms and social engagement as covariates

	Crude model				Adjusted model 1				Adjusted model 2			
	OR	95% CI	p-value	VIF	OR	95% CI	p-value	VIF	OR	95% CI	p-value	VIF
Satisfaction with meaningful activities	0.76	0.615-0.943	0.012	-	0.74	0.599-0.925	0.008	1.01	0.79	0.633-0.996	0.046	1.05

Satisfaction with meaningful activities by each question item for SMC

Table 3 presents the satisfaction levels with meaningful activities corresponding to each question item related to SMC. Among the four question items assessed, older adults who responded affirmatively to SMC-2 and SMC-4 exhibited notably reduced satisfaction with meaningful activities (SMC-2, p = 0.027; SMC-4, p = 0.004).

**Table 3:** Satisfaction with meaningful activities by each question item for SMC SD: Standard deviation; SMC: subjective memory complaints Statistical tests performed:^ †^Student’s t-test SMC-1: “Do you have any difficulty with your memory?”; SMC-2: “Do you forget where you have left things more than you used to?”; SMC-3: “Do you forget the names of close friends or relatives?”; SMC-4: “Do other people find you forgetful?”

	Sample size (n)	Satisfaction with meaningful activity, mean (SD)	Effect size	p-value^†^
SMC-1				
Yes	152	4.3 ± 1.0	0.129	0.217
No	387	4.4 ± 0.8	-	-
SMC-2				
Yes	239	4.3 ± 0.9	0.192	0.027
No	300	4.4 ± 0.8	-	-
SMC-3				
Yes	153	4.2 ± 0.9	0.164	0.104
No	386	4.4 ± 0.8	-	-
SMC-4				
Yes	66	4.1 ± 0.9	0.375	0.004
No	473	4.4 ± 0.7	-	-

## Discussion

This study examined the relationship between SMC and satisfaction with meaningful activities among community-dwelling older adults. The results revealed that SMC was associated with satisfaction with meaningful activities. Furthermore, older adults who positively responded to SMC-2 “Do you forget where you have left things more than you used to?” and SMC-4 “Do other people find you forgetful?” had significantly lower satisfaction with meaningful activities.

The prevalence of SMC in the present study was 63.2% (n = 339), which was higher than in previous studies. The wide variance in SMC prevalence is due to the different questions used in each study. Further research on evaluation methods is needed, as research on SMC is underdeveloped and consistent evaluation methods are not available. Participants with SMC showed significantly weaker memory abilities and higher prevalence of physical frailty and depressive symptoms when compared to those without SMC. These results are consistent with those of previous studies [1,24,25].

As one of its key strengths, this study reveals that SMC is related to satisfaction with meaningful activities, regardless of the category of activities. Previous studies have shown that SMC is linked to decline of household, leisure, and social activities; however, activities based on physical exercise have proven to be effective in enhancing cognitive function, yet less effective in improving SMC [6,18,26]. This discrepancy may be explained by the meaning and value people derive from this activity. According to Cantor and Sanderson, well-being is improved when individuals participate in activities that they find intrinsically valuable and have chosen autonomously [27]. Eakman found substantial connections between meaningful activities and the fulfilment of basic psychological needs [28]. Satisfaction with activity is also related to subjective well-being, and it modifies the relationship between activity and measures of well-being in older adults [29]. Thus, participation in and high satisfaction with activities that are meaningful (valuable) to the individual of his or her choosing may lead to a higher sense of well-being and fulfilment of basic psychological needs. SMC is more likely to be accompanied by depressive symptoms due to the stress of perceived memory impairment in daily life and is presumed to have unmet psychological needs. It is therefore possible that SMC may be related to lower satisfaction with meaningful activities through the mediation of lower psychological needs. However, this study did not include an assessment of psychological needs and future studies should include those assessments.

In this study, satisfaction with meaningful activities was significantly lower among older adults who positively responded to SMC-2 and SMC-4. The SMC-2 assesses an individual’s memory function through self-recall of forgetfulness in daily life, while the SMC-4 assesses not only an individual's own feelings but also the perceptions of others [30]. These two questionnaire items are considered “self-objective assessments” because they provide a retrospective, objective view of one’s state [30]. Older adults with low self-objective assessments showed decreased participation in leisure and social activities, suggesting a link between low self-objective assessments and decreased activity participation among the older adults [6]. These results suggest that changes in activity among the older adults may affect their self-objective assessments. It is possible that decreased satisfaction with even meaningful activities may lead to decreased self-objective assessments and related to SMC. Self-objective assessments require positive intervention to further increase awareness of memory impairment in older adults who complain of SMC. However, as there are a variety of contexts for the subjective cognitive functioning questions, the interpretation of these results needs to consider the meaning given to the questions.

This study has several limitations that warrant consideration in interpreting its findings. Firstly, it was a cross-sectional study, precluding establishment of causal relationships between SMC and meaningful activities, including satisfaction. Future longitudinal studies are necessary to elucidate causal pathways. Secondly, potential confounding factors, such as psychological needs, were not measured in this study. Future research could enhance the robustness of findings by including these variables. Thirdly, the SMC question items used in this study are commonly employed in Japan; however, exploring the utility of other SMC items with potentially greater biological or social relevance is recommended to deepen understanding of the SMC-meaningful activities relationship. Fourthly, data were collected exclusively from a single city, potentially limiting the generalizability of results due to local variations that influence daily meaningful activities. Lastly, the study participants were community-dwelling residents undergoing health check surveys, which may not fully represent the broader population as they were not randomly selected and may exhibit higher levels of health and survey participation motivation.

## Conclusions

This study found a relationship between SMC and satisfaction with meaningful activities among community-dwelling older adults. The results indicate that community-dwelling older adults with SMC reported a lower level of satisfaction with meaningful activities than their healthier counterparts. The examination of SMC and meaningful activities among community-dwelling older adults may provide useful information when considering health promotion strategies. However, this study only uncovered cross-sectional relationships; thus, further research is necessary to build a more substantial body of evidence.

## Appendices

**Table 4:** Four questionnaires for the SMC

Questionnaire	Response items
1. Do you have any difficulty with your memory?	a. Yes, b. No
2. Do you forget where you have left things more than you used to?	a. Yes, b. No
3. Do you forget the names of close friends or relatives?	a. Yes, b. No
4. Do other people find you forgetful?	a. Yes, b. No
